# Industry payments to physician journal editors

**DOI:** 10.1371/journal.pone.0211495

**Published:** 2019-02-07

**Authors:** Victoria S. S. Wong, Lauro Nathaniel Avalos, Michael L. Callaham

**Affiliations:** 1 Department of Medicine, John A. Burns School of Medicine at the University of Hawai‘i at Mānoa, Honolulu, Hawaii, United States of America; 2 Neuroscience Institute, The Queen's Medical Center, Honolulu, Hawaii, United States of America; 3 University of California, San Francisco, California, United States of America; the University of Sydney, AUSTRALIA

## Abstract

**Background:**

*Open Payments* is a United States federal program mandating reporting of medical industry payments to physicians, increasing transparency of physician conflicts of interest (COI). Study objectives were to assess industry payments to physician-editors, and to compare their financial COI rate to all physicians within the specialty.

**Methods and findings:**

We performed a retrospective analysis of prospectively collected data, reviewing *Open Payments* from August 1, 2013 to December 31, 2016. We reviewed general payments (“… not made in connection with a research agreement”) and research funding to “top tier” physician-editors of highly-cited medical journals. We compared payments to physician-editors and physicians-by-specialty. In 35 journals, 333 (74.5%) of 447 “top tier” US-based editors met inclusion criteria. Of these, 212 (63.7%) received industry-associated payments in the study period. In an average year, 141 (42.3%) of physician-editors received any direct payments to themselves including general payments and research payments, 66 (19.8%) received direct payments >$5,000 (National Institutes of Health threshold for a Significant Financial Interest) and 51 (15.3%) received >$10,000. Mean annual general payments to physician-editors was $55,157 (median 3,512, standard deviation 561,885, range 10–10,981,153). Median general payments to physician-editors were mostly higher compared to all physicians within their specialty. Mean annual direct research payment to the physician-editor was $14,558 (median 4,000, range 15–174,440). Mean annual indirect research funding to the physician-editor’s institution (highly valued by academic leaders such as departmental chairs and deans) was $175,282 (median 49,107, range 0.18–5,000,000). The main study limitation was difficulty identifying physician-editors primarily responsible for making manuscript decisions.

**Conclusions:**

A substantial minority of physician-editors receive payments from industry within any given year, sometimes quite large. Most editors received payment of some kind during the four-year study period. Given the extent of editors’ influences on the medical literature, more robust and accessible editor financial COI declarations are recommended.

## Introduction

The *Open Payments* program, also known as the *Physician Payments Sunshine Act*, is a federal program in the United States (US) under the Affordable Care Act that aims to increase transparency of physician conflicts of interest (COI) by providing information about medical industry payments to physicians and teaching hospitals.[1] Drug and device manufacturers must, by law, report payments to licensed physicians or teaching hospitals[2] or risk large fines if they fail to comply.[3] The *Open Payments* search tool[4] provides this data to the public, with data collection that started August 1, 2013.[2] This data should increase the accuracy of financial COI information compared to the previously reported high rate of COI nondisclosure among physicians.[5]

One group of physicians for whom COI are particularly relevant is physician journal editors who make decisions about which research manuscripts to publish, and thus play a major role in shaping medical knowledge and practice. Present nonspecific requirements regarding medical editor COI declarations by the International Committee of Medical Journal Editors stand in strong contrast to more detailed and specific requirements by the same organization for declarations by authors which must accompany each paper published.[6]

Few studies have sought to clarify the degree and types of COI among editors of medical journals, with early studies focusing on whether editor COI were disclosed at all. Smith et al. found that in 2011, only three of the top ten peer-reviewed medical journals had public declaration of editor COI.[7] Bosch et al. found also in 2011 that among high-impact medical journals, 38.8% required COI disclosure from editors.[8]

There is a paucity of studies that examine actual industry payments to medical editors, and these existing studies have been based on voluntary data from editors rather than a broad inclusive database with mandatory reporting. Wong et al. surveyed 95 editors-in-chief of clinical medical journals in 2009 and found that 9% reported receiving $1,000 or more from industry in the past year.[9] Janssen et al. reviewed disclosure statements from conferences and other sources, applying this information to editorial board members of five leading spine journals, and identified that 29% of editorial board members reported potential COI, and of those, 42% reported a financial relationship of greater than $10,000 in the previous year.[10]

Our study uses *Open Payments* data to specifically quantify financial COI among physician journal editors in prominent medical journals. We also compare financial payments to physician journal editors with existing research data on financial COI within the medical specialties of internal medicine, neurology, surgery, cardiology, psychiatry, pediatrics, and emergency medicine. We hypothesized that physician journal editors would have a non-negligible rate of financial COI though lower than the estimated rate among clinicians in the field, previously established using the same data source.[11]

## Methods

### Journal selection

Our goal in selecting journals was to aim for clinical specialties encompassing a variety of practices (e.g., primary and specialty care, interventional/surgical care) and accounting for a large proportion of directly clinically relevant recommendations and conclusions. We identified the journals in 2015 with the highest number of citations as stated by the InCites Journal Citation Reports[12] within the following seven medical categories: general/internal medicine, neurology, general surgery, cardiology, psychiatry, pediatrics, and emergency medicine. Journals were included, prior to data collection, if they were “clinically relevant” (rather than focusing on basic science research), as determined by a previously published protocol.[9] Journals were excluded by author consensus if the journal focus was not representative of the medical specialty or its subspecialties as defined by availability of a formal Accreditation Council for Graduate Medical Education (ACGME) residency or fellowship training program specific to that medical category. For example, cardiothoracic surgery and vascular surgery journals were included since those are fellowships after general surgery residency whereas journals for surgical specialties with separate training pathways such as orthopedics and plastic surgery were excluded. Journals were also excluded if 50% or more of the editors were based outside of the US, since the *Open Payments* search tool includes only physicians based in the US. Based on these inclusion and exclusion criteria, we chose the top five journals in total citations within each of the seven medical categories. A mini-Delphi technique was used among the authors to reach agreement in difficult cases.[13]

We chose to use total citations rather than journal impact factor to identify journals for review; both are differing but legitimate means to identify top journals. Journal impact factor is often used as a measure of impact, but is purely a measure of citations per journal measured over a short recent time period. A particular paper in a high impact factor journal may be read by few and sometimes by none. Total number of citations of individual papers in other journals, especially over years, may be more indicative of dissemination to (and use by) a large universe of readers and researchers.

### Editor selection

We reviewed each journal’s masthead to identify a “top tier” of physician-editors judged to be senior within the editorial hierarchy, aiming to include editors most likely to be directly responsible for making manuscript decisions (not simply comments or suggestions). Mastheads had significant variability regarding editor titles. Informal query of a number of medical editors confirmed heterogeneity of practice across journals. There was thus no editorial board category listing that precisely identified editors who made decisions about manuscript acceptance, and those who did not, nor is this information listed on editorial mastheads.

Mastheads do not clearly and systematically identify a “top tier” of physician-editors for each journal likely to be involved in manuscript decision-making. Surveys to obtain this information directly from editors and/or journals have had unreliably low response rates.[14] Therefore we sought to identify editors in a decision-making role by a set of inclusion/exclusion criteria:

- Include the editor-in-chief
- Include editors with titles containing “deputy,” “senior,” “executive,” “head,” “vice,” or “associate,” UNLESS they also have “assistant” in their title
- Exclude all editors with “assistant” in their title
- Exclude all “consulting” and “managing” editors
- Exclude all section or “specialty” editors UNLESS there are no higher-ranking editors (besides editor-in-chief) in which case all clinical section editors are included
- Exclude statistical, epidemiological, CME, and other non-clinical editors
- Exclude all non-US regional editors
- Exclude the “editorial board” or other similar “hanging committee” of consulting editors or committee members, UNLESS there are no higher-ranking editors (besides editor-in-chief) in which case the entire editorial board is included
- Exclude editors emeritus, guest editors, advisory editors
- Exclude non-physician editors
- Exclude physicians based at institutions outside the US

### Outcome measures

For each physician-editor fulfilling inclusion criteria, we collected all available payments data using the *Open Payments* search tool. August 1, 2013 to December 31, 2015 data were collected prior to the June 30, 2017 data release, whereas January 1, 2016 to December 31, 2016 data were collected after the June 30, 2017 data release.[15] Collected data categories are defined by the database managers and included:[15–17]

1) Total general payments from industry (defined as “Payments or other transfers of value not made in connection with a research agreement”),

2) Total “direct” research payments (defined as “Payments where the company making the payment has named a physician as the primary recipient”), and

3) Associated “indirect” research funding (defined as “Payments to a research institution or entity where a physician is named as a principal investigator on the research project”).

We also collected data on any reported industry ownership or investment.[15] We excluded disputed payments flagged by physician recipients as being incorrect data.

Since the *Open Payments* database does not include physicians that have no industry payments, all identified editors had their physician degrees (MD, DO, or international equivalent) confirmed using a Google search to establish an accurate denominator. We used a recent observational retrospective study focusing on industry payments to physicians by specialty for comparison data.[11]

Simple descriptive statistics were used for data analysis.

### Institutional review board

This study was reviewed and approved by The Queen’s Medical Center Research & Institutional Review Committee, and qualified as exempt under the University of California, San Francisco Institutional Review Board.

## Results

**Fig 1:**
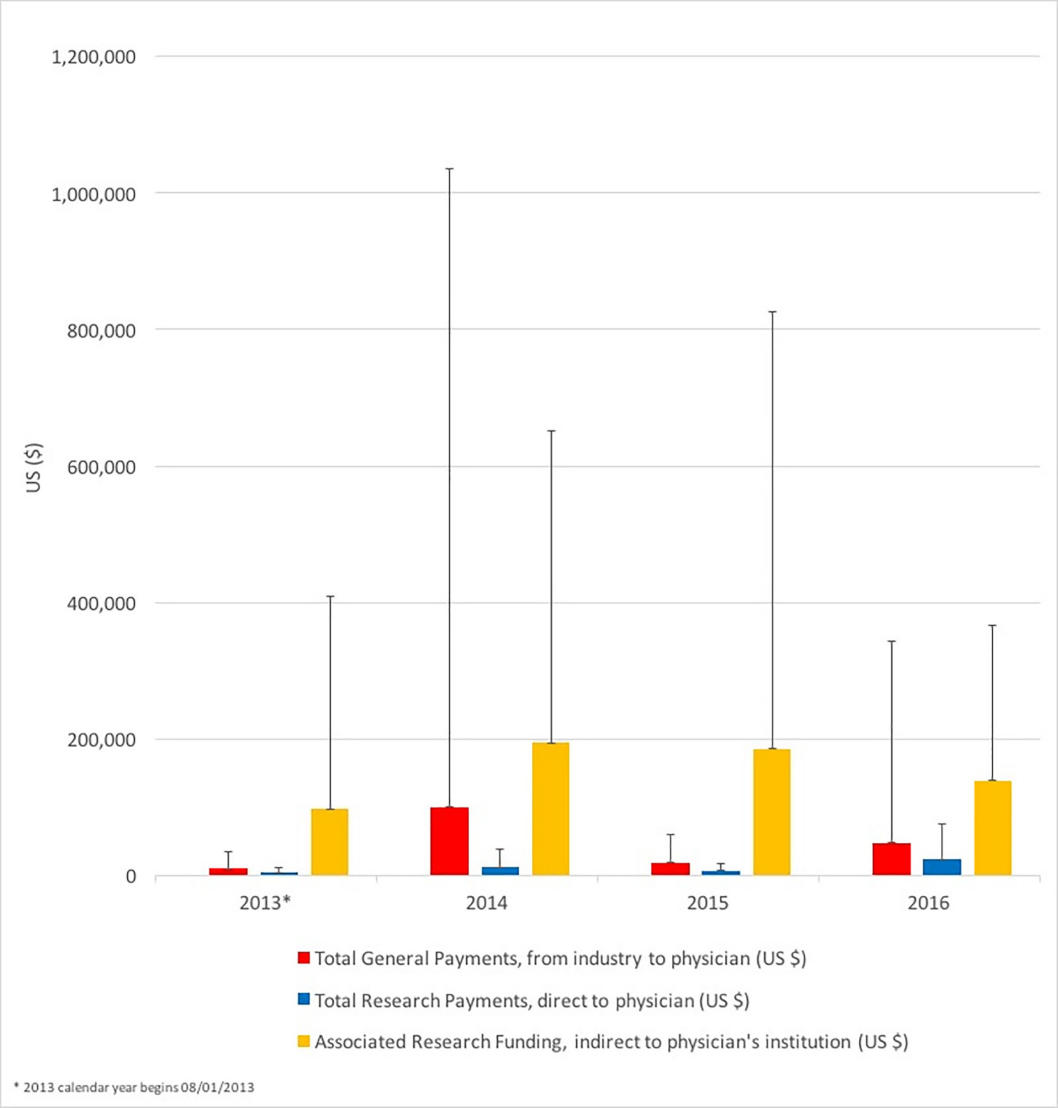
Mean industry payments to physician-editors by year with standard deviations.

Out of 447 “top tier” editors of 35 journals, 333 (74.5%) met inclusion criteria as physician-editors based at a US institution (mean: 9.5 editors per journal; standard deviation: 7.4; range: 1–30). Of these, 212 (63.7%) physician-editors received industry-associated payments of any kind in the 41-month period. Averaging complete (12-month) 2014 to 2016 data, 139 (41.7%) physician-editors received general payments from industry per year, 19 (5.7%) received total “direct” research payments, and 60 (18.0%) received associated “indirect” research funding. Disputed payments represented a very small proportion of all transactions, with only 20 (0.1%) transactions (out of 14,101) from 7 individuals totaling $138,152.76 over the 41-month period, and were excluded. Mean industry payments among physician-editors receiving payments are separated by type and year in Fig 1.

Total payments of each type (total general payments, direct total research payments, and indirect associated research funding) averaging complete 2014 to 2016 data are detailed in Table 1 below. For each payment type, analyses were performed for all qualifying physician-editor recipients of each payment type (i.e., physician-editors with non-zero *Open Payment* entries). Editors-in-chief were analyzed separately. A total of 35 editors-in-chief from 32 unique journals met inclusion criteria. (Three journals each had two editors-in-chief included who were given “equal billing” on the masthead.) As a form of further sensitivity testing, for each payment type, analyses were performed for all qualifying physician editors-in-chief recipients of each payment type (i.e., physician editors-in-chief with non-zero *Open Payment* entries).

**Table 1:** Annual payments to physician-editors and editors-in-chief using combined 2014 to 2016 data.

	Annual mean n (%) receiving any payment	Annual mean, US $	Annual standard deviation, US $	Annual range, US $	Annual median (IQR), US $	3-year total (2014 to 2016 US $)
**Total general payments to physician-editors**	139 (42)	55,157	561,885	10–10,981,153	3,512 (135–20,000)	23,000,595
**Total (direct) research payments to physician-editors**	19 (6)	14,558	34,471	15–174,440	4,000 (1,050–10,000)	829,824
**Associated (indirect) research funding to physician-editors**	60 (18)	175,282	479,480	0.18–5,000,000	49,107 (12,543–130,947)	31,550,838
**Total general payments to physician editors-in-chief**	14 (40)	9,598	14,322	12–64,318	4,111 (396–12,050)	412,713
**Total (direct) research payments to physician editors-in-chief**	1 (3)	3,913	124	3,825–4,000	3,913 (3,869–3,956)	7,825
**Associated (indirect) research funding to physician editors-in-chief**	5 (14)	214,343	352,010	5,302–1,350,000	110,718 (35,628–168,090)	3,000,807

Abbreviation: IQR, interquartile range.

Reported industry ownership and investment transactions were rare though prominent. The largest amount was a $12,736,276 declaration of stock ownership, held by an immediate family member. This made up the bulk of a total of $12,766,532 in three transactions reported over the 41-month period, all by a single individual.

**Fig 2:**
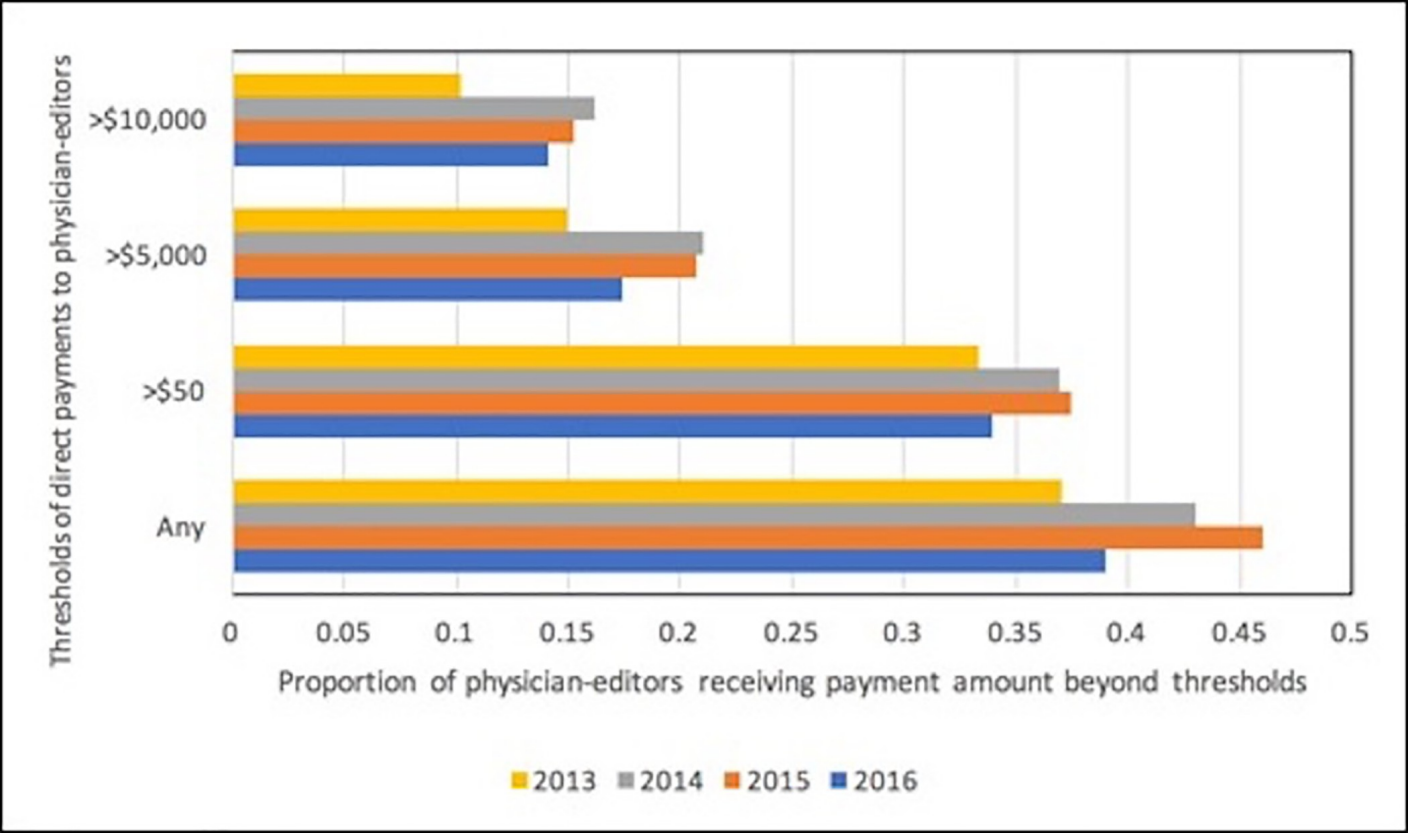
Proportion of physician-editors receiving payments directed to themselves beyond various arbitrary thresholds, 2013 to 2016.

In order to analyze payments directly benefiting the editors, we performed a separate analysis combining the “general payments” and “total direct research payments” categories, but excluding the associated “indirect” research funding category in which payments were for the research institution. During the 41-month period, 100 (30.0%) editors received payments directed to themselves, not to their institution, of >$5,000 within a year. The threshold of $5,000 is designated by the National Institutes of Health (NIH) as a Significant Financial Interest (SFI).[18] Averaging 2014 to 2016 data, on an annual basis, 141 (42.3%) of physician-editors received any payments directed to themselves rather than their institution, 120 (36.0%) received payments >$50; 66 (19.8%) received payments >$5,000; and 51 (15.3%) received payments >$10,000. Thresholds by year are identified in Fig 2.

Using the same data source, general payments received by physician-editors of specialty journals were compared to all physicians within that specialty, using data from a previously published study for the latter values in Table 2.[11]

**Table 2:** “Per-Physician” general payments received by physician-editors versus all physicians within a specialty in 2015^a^.

Specialty	Physician-editors			All physicians within specialty[11]		
	Number of recipients (%)	Median value (IQR), US $	Number (%) receiving >$10,000	Number of recipients (%)	Median value (IQR), US $	Number (%) receiving >$10,000
Cardiology	50 (76)	17,704 (2,430–38,096)	29 (44)	22,044 (75)	862 (226–2,749)	2,661 (12)
Emergency Medicine	16 (25)	114 (66–9,141)	4 (6)	12,733 (25)	50 (18–125)	167 (1)
Internal Medicine	17 (25)	5,052 (91–31,391)	7 (10)	103,588 (51)	248 (73–959)	5,167 (5)
Neurology	10 (29)	207 (65–2,655)	0 (0)	10,794 (60)	541 (125–2,120)	1,275 (12)
Pediatrics	8 (36)	1,518 (25–3,887)	1 (5)	33,536 (40)	94 (32–203)	559 (2)
Psychiatry	16 (46)	9,016 (3,332–16,686)	8 (23)	19,922 (37)	171 (58–539)	722 (4)
General Surgery	33 (79)	444 (63–1,310)	1 (2)	21,857 (56)	251 (81–1,112)	990 (5)

Abbreviation: IQR, interquartile range.

^a^ Physician-editors not receiving general payments were excluded from analysis

## Discussion

The *Open Payments* federal program has, for the first time, allowed for reasonably complete transparency in financial COI among US physicians. In this study, we focused on COI among physician-editors at the top of the editorial hierarchy. A substantial minority of physician-editors receive payments from industry within any given year. However, over the 41-month study period, physician-editors directly or indirectly receiving industry payments of some kind represented a majority. In some cases, physician-editors have very large COI. Also, median general payments to physician-editors are mostly higher compared to all physicians within the specialty suggesting at the very least that physician-editors do not have a lesser degree of COI compared to their non-editor peers. This was an unexpected finding since journal editing is typically perceived as a non-commercial endeavor performed by academic physicians, preferably based on scientific evidence rather than other biases.

One other study by Liu et al. examined industry payments to physician-editors as well, focusing specifically on 2014 *Open Payments* data rather than the 2013 to 2016 period as with our present study.[14] The Liu et al. study examined editors of the top two journals from each of 26 medical disciplines whereas our study examined editors of the top five journals from each of seven disciplines. Their study found that in 2014 amongst their study sample, 50.6% of physician-editors received general payments whereas our study found that 41.7% of physician-editors received general payments. Although a direct comparison cannot be made due to the different journals reviewed, the two numbers are comparable despite the wider breadth of the Liu et al. study and the longer study period covered by our study. This is also similar to the approximately 48% of United States physicians who received industry payments in 2015.[11] Liu et al. also reviewed 52 journal websites, noting that editor conflict-of-interest policies were readily accessible for 32.7% of reviewed journals. Their study chose journals to review based on impact factor, whereas we chose journals based on total citations.

Additionally and importantly, our study included both type of payments for research support provided in the database: directly to individual physicians, or to research institutions (rather than to a named physician). The latter was a category excluded from the BMJ study. We believe this category, which was larger than any of the others, was important to include. Although the physician-editor does not directly receive this money personally, it usually remains very beneficial to them (since the institution frequently uses it for their salary support and research expenses.) These funds are also valued by department chairs, deans, and the receiving institutions, and increase the prestige and influence of the editor-recipient, thus creating an important potential source of bias.

Although the NIH has currently designated a $5,000 threshold as being a SFI (previously defined as $10,000),[18] we were unable to identify any objective data supporting this amount. It is known that even small payments can result in bias. For example, among prescribing physicians, a single meal promoting a brand-name medication results in an increased rate of prescribing that medication.[19] Readers will have to reach their own conclusions whether a large number of small payments to editors making decisions, or a small number of very large ones, is most likely to introduce bias, but it seems inescapable that bias would be introduced to some degree, and that currently a reader cannot know how much or by whom.[20]

Our study had limitations. Though we aimed to target physician-editors primarily responsible for making manuscript decisions, we were unable to confirm each editor’s role, nor we could confirm whether editors worked part- or full-time in paid or unpaid editorial positions. Also, using our inclusion criteria for identifying handling editors within a journal’s masthead led to significant variability in the number of editors reviewed per journal. However, no standard system exists for distribution or identification of editorial roles, so we chose a conservative definition to focus attention on editors most likely to make manuscript decisions. Our analysis on editors-in-chief focused even more specifically on the group of editors with the highest level of decision authority within a journal who most likely participated in all acceptance decisions.

There have also been critiques of the *Open Payments* database in the past, including the vagueness of payment categories, low rates of physician and institutional review of data for accuracy, unresolved disputed records, and a paucity of contextual commentary.[3,21] Data collection occurred entirely in 2017 for this present study, bypassing earlier issues with missing data.[22]

Beyond deciding what gets published in medical journals (thereby shaping published research and widespread clinical practice), journal editors also decide who the peer reviewers are, which ones to use for a particular article, which articles are prioritized within a journal issue, and also determine the need for additional editorials or commentary, which might be pro or con the article’s conclusions. Presently at most journals, authors and reviewers are required to declare COI in detail, meaning that this precaution is expected for everyone involved in the peer review and publication process except the editors who make the key decisions. In any other setting, such a position would be considered to be very vulnerable to COI and perhaps the most important step in the process of preventing COI. At most journals, however, there is not a standardized approach or requirement for how or whether financial COI are reported to readers. Some journals have established criteria for editor recusal,[23] though such formal recusal policies are not wide-spread.

Standards for declaration of financial COI among medical editors are presently poorly-defined in comparison to the vigorous requirements for declaration of COI by manuscript authors and CME presenters. Requiring full transparency in declaring financial COI among medical editors may be a reasonable first step in identifying potential conflicts, though one could argue that mere transparency is not sufficient.[24] Financial COI have been shown to result in biased behavior within biomedical and clinical research,[25–27] and also among prescribing physicians.[28,29] Since editorial decisions are a black box, it is difficult to determine the explicit reasons why an editor makes a manuscript decision and cognitive research suggests that they might very well not be conscious of the reasons themselves,[30] so any amount of financial COI, even declared, may result in unacceptable bias.

Based on our study results that most editors are exposed to some direct financial COI, we recommend at the very least, a system of mandatory financial COI declaration for medical editors similar to that required of authors (and ideally, of proven efficacy). Listing the COI of all editors somewhere in the journal cannot help the reader unless each published article names all editors who were involved in handling and decision-making for that particular manuscript, and declares whether they had COI related to the published study. This information could be published linked to the article itself for maximal transparency, and perhaps following the current International Committee of Medical Journal Editors (ICMJE) specific format that is already mandatory for authors. Finally, the most definitive but also disruptive and probably impractical remedy would be for editors to avoid taking any industry funds at all, removing this source of bias entirely.
